# Physicians’ Visiting List for 1884

**Published:** 1884-01

**Authors:** P. Blakiston

**Affiliations:** Son & Co., Philadelphia


### Physician's Visiting List for 1884.

P. Blakiston, Son & Co.,Philadelphia.

This very complete little *Engagement* an l *List Manuel* for 1884 has just come to hand. It is primarily for the physician's use, but can be used by the specialist as well. It has a calender for 1884–5; gives methods of treatment in asphyxia; poisons and antidotes; metric system of weights and measures; a dose table, which is almost invaluable for ready reference; also, a list of the most important new remedies, and a few old ones which have been recently revived.

Taken as a whole this is the most perfect memorandum ever issued for the purpose. This is the thirty-third year of its issue, and the aim all these years has been to reach the best thing. It may be obtained of Robert Clark & Co., of Cincinnati, and of book sellers generally. It is made of various sizes, varying in price from $1.00 to $3.00.

